# Characterization of two novel VIM-type metallo-β-lactamases, VIM-84 and VIM-85, associated with the spread of IncP-2 megaplasmids in *Pseudomonas aeruginosa*

**DOI:** 10.1128/spectrum.01544-23

**Published:** 2023-09-14

**Authors:** Nanfei Wang, Tailong Lei, Yiwei Zhu, Yue Li, Heng Cai, Piaopiao Zhang, Sebastian Leptihn, Junxin Zhou, Huanhuan Ke, Bo Gao, Yu Feng, Xiaoting Hua, Tingting Qu

**Affiliations:** 1 State Key Laboratory for Diagnosis and Treatment of Infectious Diseases, National Clinical Research Center for Infectious Diseases, National Medical Center for Infectious Diseases, Collaborative Innovation Center for Diagnosis and Treatment of Infectious Diseases, The First Affiliated Hospital, Zhejiang University School of Medicine, Hangzhou, Zhejiang, China; 2 Department of Infectious Diseases, Sir Run Run Shaw Hospital, Zhejiang University School of Medicine, Hangzhou, China; 3 Key Laboratory of Microbial Technology and Bioinformatics of Zhejiang Province, Hangzhou, China; 4 Regional Medical Center for National Institute of Respiratory Diseases, Sir Run Run Shaw Hospital, Zhejiang University School of Medicine, Hangzhou, China; 5 Department of Biochemistry, Health and Medical University, Erfurt, Germany; 6 Department of Antimicrobial Biotechnology, Fraunhofer Institute for Cell Therapy & Immunology, Leipzig, Germany; 7 Department of Biophysics, and Department of Infectious Disease of Sir Run Run Shaw Hospital, Zhejiang University School of Medicine, Hangzhou, China; 8 Alibaba-Zhejiang University Joint Research Center of Future Digital Healthcare, Hangzhou, Zhejiang, China; Universidad de Buenos Aires, Buenos Aires, Argentina

**Keywords:** *Pseudomonas aeruginosa*, metallo-β-lactamase, VIM, megaplasmid, antimicrobial resistance

## Abstract

This study aimed to characterize two novel VIM-type metallo-β-lactamases, VIM-84 and VIM-85, and reveal the important role of the IncP-2 type megaplasmids in the spread of antimicrobial resistance (AMR) genes. VIM-84 and VIM-85 were encoded by two novel genes *bla*_VIM-84_ and *bla*_VIM-85_ which showed similarity to *bla*_VIM-24_. Both *bla*_VIM-84_ and *bla*_VIM-85_ are harbored into class 1 integrons embedded into the Tn*1403* transposon. The *bla*_VIM-85_ gene was identified in a megaplasmid, which was related to 17 megaplasmid sequences with sizes larger than 430 kb, deposited previously in Genbank. A comparative analysis of complete plasmid sequences showed highly similar backbone regions and various AMR genes. A phylogenetic tree revealed that these megaplasmids, which were widely distributed globally, were vehicles for the spread of AMR genes. The *bla*_VIM-24_, *bla*_VIM-84_, and *bla*_VIM-85_ genes were cloned into pGK1900, and the recombinant vectors were further transformed into *Escherichia coli* DH5α and *Pseudomonas aeruginosa* PAO1. The antimicrobial susceptibility test of the cloning strains showed high levels of resistance to β-lactams while they remained susceptible to aztreonam. Enzymatic tests revealed that both, VIM-84 and VIM-85, exhibited higher activity in hydrolyzing β-lactams compared to VIM-24. A D117N mutation found in VIM-24 affected binding to the antibiotics.

**IMPORTANCE:**

The metallo-β-lactamases-producing *Pseudomonas aeruginosa* strains play an important role in hospital outbreaks and the VIM-type enzyme is the most prevalent in European countries. Two novel VIM-type enzymes in our study, VIM-84 and VIM-85, have higher levels of resistance to β-lactams and greater hydrolytic activities for most β-lactams compared with VIM-24. Both *bla*_VIM-84_ and *bla*_VIM-85_ are harbored into class 1 integrons embedded into the Tn*1403* transposon. Notably, the genes *bla*_VIM-85_ are carried by three different IncP-2-type megaplasmids which are distributed locally and appear responsible for the spread of antimicrobial resistance genes in hospital settings.

## INTRODUCTION

Increasing carbapenem-resistant *Pseudomonas aeruginosa* (CRPA) has become a concerning health issue worldwide (1). Enzymatic mechanisms of carbapenem resistance, especially the production of β-lactamases, are essential in *P. aeruginosa*. The class B β-lactamases are zinc-dependent, which are known as metallo-β-lactamases (MBLs) (2), and possess the capability to hydrolyze all β-lactams including carbapenems and β-lactam/β-lactamase inhibitor combinations (3). MBLs-producing *P. aeruginosa* strains are responsible for many hospital outbreaks across the world. The VIM-type enzyme is the most prevalent in European countries and over 80 variants have been identified so far (4). Routine amino acid point substitutions are most common in novel variants resulting in distinct functional differences (5). The *bla*_VIM_s are commonly embedded in genetic cassettes, often part of a variety of class 1 integrons that are inserted into transposons, mediating widespread dissemination of the genes (6). The genes encoding MBLs are also often found in transferable plasmids which have a narrow host range and are classiﬁed by a separate incompatibility typing system. These plasmids are generally categorized as IncP-2 group and have a size of >350 kb, thus aptly called megaplasmids (7).

In this study, we report the identification and characterization of two new variants of VIM-24, VIM-84 and VIM-85, which were identified in *P. aeruginosa* strains isolated during a multicenter surveillance study in China. Notably, the resistance gene *bla*_VIM-85_ was detected in megaplasmids from three clinical CRPA strains.

## MATERIALS AND METHODS

### Bacterial strain collection and identification

Five clinical CRPA isolates named WTJH2, WTJH6, WTJH32, WTJH36, and WTJH43 were recovered from bronchoalveolar lavage samples of patients hospitalized in the vascular surgery department of Wuhan Tongji Hospital (WTJH) in 2018. The strains were cultured on the Pseudomonas Isolation Agar plates (Peptone 20 g/L, MgCl 21.4 g/L, K_2_SO_4_ 10 g/L, Triclosan 25 mg/L, Agar 13.6 g/L, and Glycerol 20 mL/L) and further confirmed by the matrix-assisted laser desorption ionization-time of flight mass spectrometry (Bruker Daltonik GmbH, Bremen, Germany) and 16S rRNA gene sequencing. The variants of the *bla*_VIM_ genes were identified by the long-read sequencing (see below) and reconfirmed by PCR ampliﬁcation.

### Whole-genome sequencing and plasmid analysis

Genomic DNA was extracted using the QIAamp DNA Mini Kit (Qiagen, Hilden, Germany) according to the manufacturer’s instructions. Sequencing was performed on the Illumina HiSeq X10 platform (San Diego, CA, USA) and Nanopore MinION long-read sequencing (Oxford Nanopore Technologies, Oxford, UK). Both short and long reads were *de novo* hybrid assembled using Unicycler v0.4.8 (https://github.com/rrwick/Unicycler) or assembled long reads by Unicycler first and further polished by Illumina short reads via pilon v0.24 (8). The assembled sequences were then annotated with Prokka 1.14.6 (https://github.com/tseemann/prokka) and manually reviewed through BLASTN/BLASTP (9). Multilocus sequence typing (MLST) was identified with MLST v2.19.0 (https://github.com/tseemann/mlst). Resistance gene typing was performed using Resfinder (10). Transposon Registry (11), ISﬁnder (https://www-is.biotoul.fr), and BacAnt (12) were utilized for the identification of mobile genetic elements. The circular genome comparison was completed using the BRIG software v.0.95 (13). The polished pangenomes were produced by the Panaroo pipeline (14) and then the maximum-likelihood unrooted phylogenetic tree was constructed by IQ-TREE (15) and visualized using iTOL v6.1.1 (16).

### Cloning

The *bla*_VIM-84_ gene with its upstream predicted promoter identified by Softberry (http://www.softberry.com) was amplified from the clinical isolate WTJH2. Since the region upstream of *bla*_VIM-85_ was not identical to that of *bla*_VIM-84_, we obtained the entire sequence of *bla*_VIM-85_ through site-directed mutagenesis to evaluate the single Asp117Asn substitution from *bla*_VIM-84_. The *bla*_VIM-24_ gene was also mutated from *bla*_VIM-84_ resulting in amino acid mutation at position 60 from Gln to Arg. The site-directed mutagenesis primers are listed in Table S1. The PCR products were recombined and cloned into pGK1900 plasmids as described previously (17), and the resulting expression vectors were subsequently introduced into *Escherichia coli* DH5α. Then the recombinant plasmids were isolated, and electroporation was used to introduce the episomal elements into *P. aeruginosa* PAO1.

### Antimicrobial susceptibility determination

The minimum inhibitory concentrations (MICs) of antibiotics were detected using the broth microdilution method. Antibiotics included: ampicillin; piperacillin; cefepime; ceftazidime; piperacillin-tazobactam; ceftazidime-avibactam; imipenem; meropenem; aztreonam; amikacin; gentamicin; levofloxacin; and colistin. *E. coil* ATCC 25922, *P. aeruginosa* strain ATCC 27853 and *Klebsiella pneumoniae* ATCC 700603 were used as the quality control. The diluted culture was incubated at 37°C overnight. The results were interpreted according to the 2022 Clinical and Laboratory Standards Institute guidelines (18).

### Conjugation and conjugation frequency

Conjugation experiments were performed with clinical isolates as the donors and a rifampin-resistant derivative of *P. aeruginosa* PAO1 as the recipient. Transconjugants were selected with rifampicin (300 µg/mL) and meropenem (4 µg/mL). The colonies of donor and recipient bacteria were cultured in 2 mL Luria Bertani (LB) medium and shaken at 37°C for 4 h. The donor and recipient strains in LB were mixed at a 1:1 ratio (100 µL, respectively), and 20 µL of the mixture was pipetted onto a sterile and 0.22-μm-pore-size Millipore filter on a Mueller-Hinton (MH) agar plate, and then cocultured at 37°C overnight. The bacterial lawn on the filter was harvested and resuspended in 200 µL LB broth, and plated onto the plate containing antibiotics that select for transconjugants at 37°C overnight. The growing colonies on the plate were conﬁrmed via PCR ampliﬁcation. The conjugation frequency is the number of transconjugants divided by the number of donors.

### Plasmid stability

The stability of the megaplasmids pWTJH36 and pWTJH43 was determined by culturing the transconjugants PAO1/pWTJH36 and PAO1/pWTJH43 through five successive generations without antibiotics. Briefly, three colonies of the transconjugant were selected as biological replicates and cultured in 3 mL LB broth without antibiotics at 37°C with shaking overnight. About 3 µL of each bacterial fluid was added to 3 mL LB broth (1:1,000 dilution), and then incubated at 37°C in a shaking incubator. The above procedure was repeated until Day 5 and 10 µL of each bacterial fluid was pipetted into 90 µL sterilized 0.9% (wt/vol) NaCl as the 10^−1^ dilution. The serial dilution process was repeated until a 10^−8^ dilution was obtained. Each clone was diluted three times independently. About 10 µL of the 10^−1^ to 10^−8^ dilutions were pipetted onto MH agar plates and antibiotics-resistant plates (rifampicin 300 µg/mL and meropenem 4 µg/mL, respectively), and all plates were incubated at 37°C overnight. The number of colony-forming units on MH agar plates and antibiotics-resistant plates was calculated and 30 clones of each transconjugant on antibiotics-resistant plates were selected for PCR verification. The primers for *bla*_VIM-85_ and *repA* are listed in Table S1.

### Plasmid fitness cost

A noncompetitive growth kinetics analysis was conducted to evaluate the ﬁtness cost of the megaplasmids pWTJH36 and pWTJH43 in PAO1 compared to plasmid-free PAO1. Three colonies of each strain were selected as biological replicates and cultured independently in 2 mL LB broth overnight. Each bacterial broth was diluted to 1:100 in MH broth and 200 µL diluted culture was pipetted into a 100-well plate in three replicates. A Bioscreen C Analyzer (Oy Growth Curves Ab. Ltd., Finland) was used to record the optical density at 600 nm (OD_600_) of each culture every 5 min at 37°C for 20 h. The growth rate based on the OD_600_ curves was calculated using an R script and the statistical analysis was performed using GraphPad Prism v9. Ordinary one-way analysis of variance multiple comparisons were used to evaluate the differences between the means. *P* values of <0.05 were considered to be indicative of a statistically signiﬁcant result.

### Expression and puriﬁcation of proteins

The open reading frames (ORFs) coding for VIM-2, VIM-36, VIM-24, VIM-84, and VIM-85 without signal peptide regions were ampliﬁed using the primers listed in Table S1. The *bla*_VIM-2_ gene was amplified from WTJH52, a clinical strain carrying *bla*_VIM-2_ isolated from this surveillance study. The *bla*_VIM-36_ gene was mutated from *bla*_VIM-84_ resulting in amino acid mutation at position 205 from Leu to Arg. The site-directed mutagenesis primer is listed in Table S1. The PCR products were then cloned into pET-28a with an N-terminal His6-Tag. The resulting recombinant plasmids were introduced into *E. coli* BL21 (DE3) by chemical transformation. *E. coli* BL21 (DE3) carrying the expression vectors were cultured in an LB medium containing 50 mg/L kanamycin until the OD_600_ reached 0.6–0.8 and the expression of the cells was induced with 1 mM IPTG overnight at 18°C with shaking, in the presence of 20 mM ZnCl_2_. Cells were harvested and resuspended in 50 mM Tris-HCl (pH 7.5) containing 200 mM NaCl and 50 µM ZnCl_2_ and then homogenized by a continuous flow homogenizer (JNBIO, China). The supernatant was purified using a Nickel-affinity-column and eluted in a linear imidazole gradient (20–300 mM). To remove impurities, the protein was further puriﬁed and the purity assessed as described previously (17).

### Determination of kinetic parameters

Substrate hydrolysis rates were determined using a D8 UV-visible spectrophotometer (Runqee, Shanghai, China) at room temperature. Assays were performed in buffer phosphate-buffered solution (1×) supplemented with 50 µM ZnSO_4_. Piperacillin, cefepime, imipenem, and meropenem were used as substrates. The extinction coefficients were: piperacillin, Δ_ε235_ = −820 M^−1^ cm^−1^; cefepime, Δ_ε260_ = −10,000 M^−1^ cm^−1^; imipenem, Δ_ε300_ = −9,000 M^−1^ cm^−1^; and meropenem, Δ_ε300_ = −6,500 M^−1^ cm^−1^ (19). The data were analyzed by GraphPad Prism v9.0.0 using the Michaelis–Menten Equation.

### Structural modeling and molecular docking

The inhibitor bounded crystal structure of the VIM-2 metallo-β-lactamase (PDB entry: 2YZ3) was used as the structure template to build the homology model of VIM-24, VIM-84, and VIM-85. *Protein Preparation Wizard* (20) in Schrödinger Release 2018-1 was used to preprocess and refine the protein structure. OPLS3e force field was used for restrained energy minimization (21). The proteins were further assessed to ensure no steric hindrance issues or other deviations. After that, the centroid of the inhibitor in the enzyme structure was set as the center of grid box, and then prepared ligands were docked there.

## RESULTS

### Drug-resistance profiles and genomic characterization of the bacterial strains

Antimicrobial susceptibility tests showed that all five clinical isolates, WTJH2, WTJH6, WTJH32, WTJH36, and WTJH43, were resistant to all tested β-lactams as well as gentamicin, but susceptible to aztreonam, amikacin, levofloxacin, and colistin (Table S2). According to the whole genome sequence analysis of the five isolates, several acquired antibiotic resistance genes were identified. All strains also contained one plasmid each, which contained novel variants of the *bla*_VIM_ genes, namely *bla*_VIM-84_ and *bla*_VIM-85_ (Table S2).

The plasmids pWTJH2 and pWTJH32, which contained the *bla*_VIM-84_ genes, had a length of ~100 kb and were found to be closely related to each other with 100% nucleotide similarity across the entire sequence. The genetic environment of the *bla*_VIM-84_ genes was identical and genes encoding an efﬂux pump (MexCD-OprJ) were detected in both plasmids. A pairwise comparison of pWTJH2 and pWTJH32 indicated a horizontal flip mediated by transposon Tn*5563a*, a transposon encoding putative mercuric ion transport proteins (22). The flipped genetic element in pWTJH32 was flanked by two copies of Tn*5563a* transposons, while pWTJH2 lost one of them (Fig. S1).

The plasmids pWTJH6, pWTJH36, and pWTJH43, the carriers of the *bla*_VIM-85_ genes were all much larger with a length of over 420 kb. Due to their size, the episomal elements could be considered megaplasmids. According to BLASTn searches, the complete sequences of pWTJH36 and pWTJH43 were highly similar with 100% coverage and 99.99% nucleotide identity. Similarly, pWTJH6 and pWTJH43 shared 98% query coverage with 99.98% nucleotide identity. Homology searches targeting the complete sequence of pWTJH43 identified 17 megaplasmids, deposited in the NCBI database, ranging in size from 394 to 513 kb with >80% coverage (Table S3).

### Genomic features of the pWTJH megaplasmids

**Fig 1:**
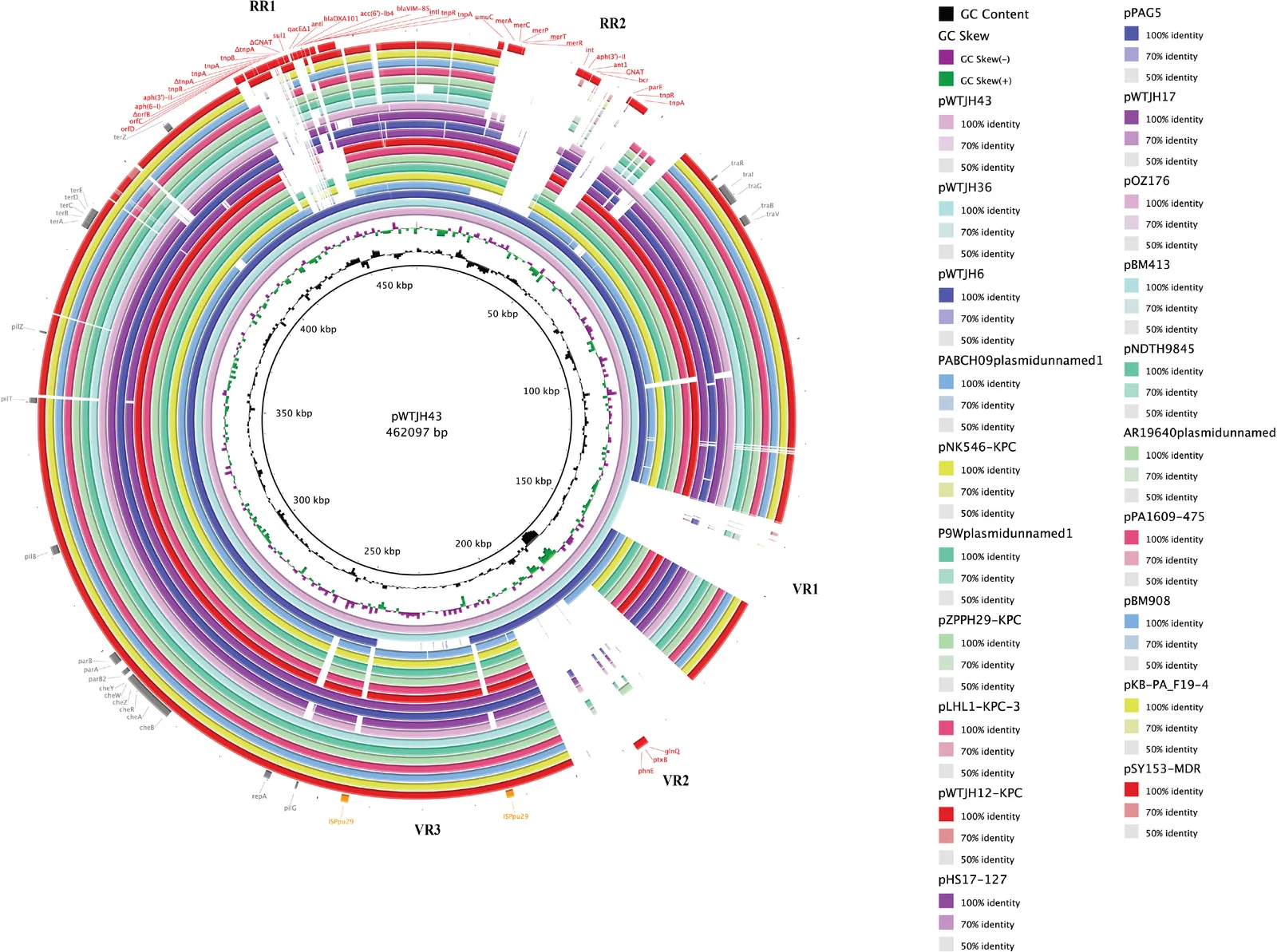
Comparison of the three pWTJH megaplasmids (pWTJH6, pWTIH36, and pWTJH43) and 17 highly related megaplasmids in Genbank. The plasmid pWTJH43 is used as a reference. The first and second circles illustrate the GC content and GC skew. The plasmids are represented by various colorful circles from the inner to the outer and the annotation of pWTJH43 is on the outmost circle.

A comparative analysis of 17 complete plasmid sequences from the NCBI Genbank and the three pWTJH megaplasmids from our study revealed highly similar genetic structures in the backbone and key elements (Fig. 1). The replication initiator RepA proteins encoded by the three pWTJH megaplasmids were identical to the one encoded in plasmid pOZ176 (located between 109,483 and 120,876 nt), which had been identiﬁed to belong to the IncP-2 group using incompatibility testing (23). This indicated that the pWTJH megaplasmids might group in the same IncP-2 incompatibility group. However, a large fragment of the *repA-oriV-parAB* region of pOZ176 was absent in the pWTJH megaplasmids, as with the IncP-2 plasmid sublineage associated with dissemination of *bla*_IMP-45_ (24). Similarly, the genes coding for the putative partitioning proteins ParA and ParB were also identical in these 20 IncP-2 plasmids. Tellurite resistance genes (*terABCDEZ*) were also identified to be part of the core backbone, shared among the plasmids. Virulence factors were also detected; these included *pilB*, *pilG*, *pilT*, and *pilZ* genes which encoded a type IV pilus (T4aP) modulating twitching motility and a chemotaxis operon (*cheBARZWY*) required for pilus assembly (25). The conjugative transfer operon *traG* gene was found and the *traI* gene, which might serve as a relaxase, was involved in initiating DNA transfer (26).

**Fig 2:**
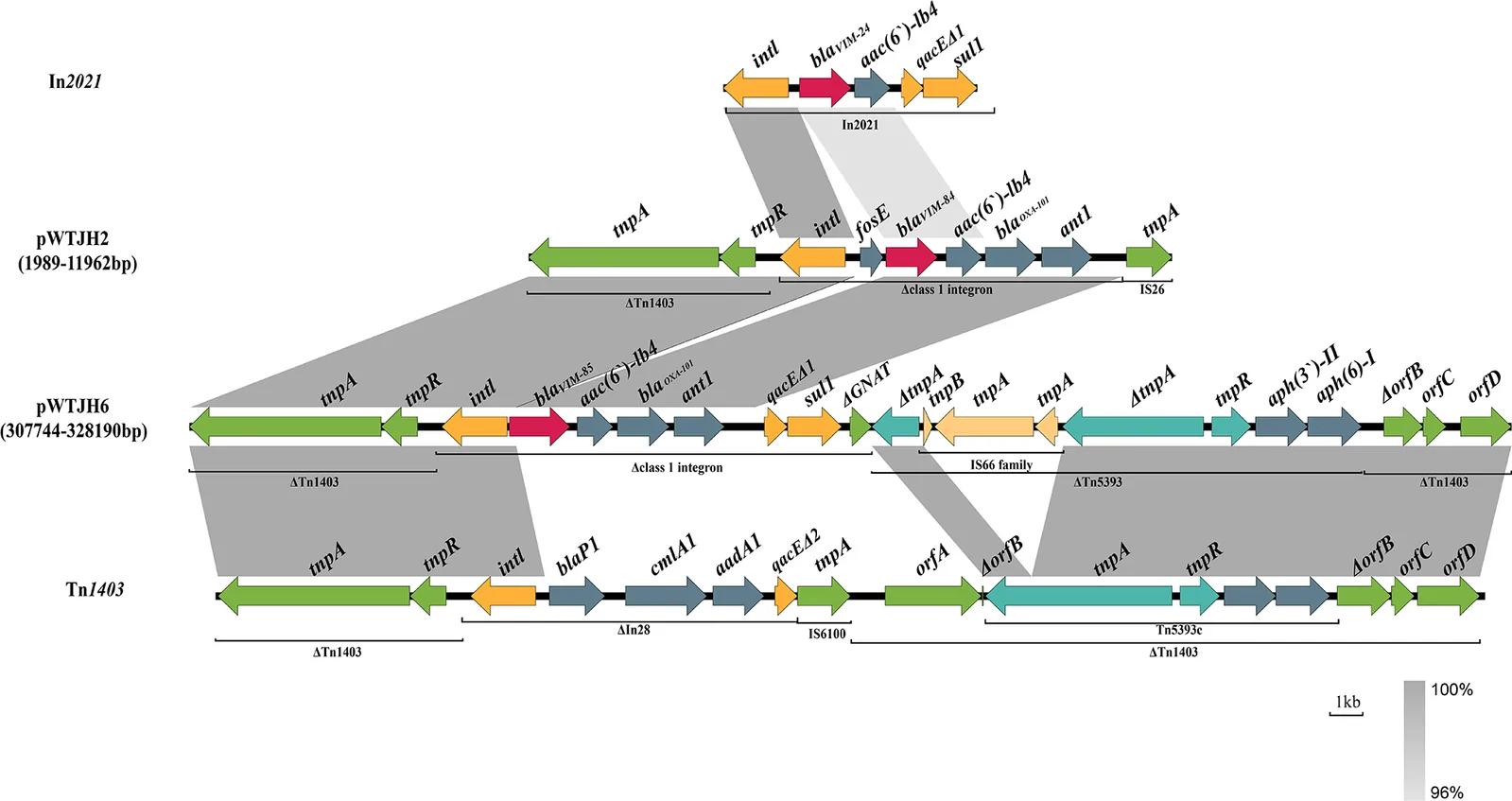
The pairwise comparisons of the genetic environment of the *bla*_VIM_. Shaded regions denote nucleotide identity (96–100%). Red arrows denote the genes *bla*_VIM_ and the other antibiotic resistance genes are denoted by dark blue arrows. Yellow, green and light blue arrows denote structures of mobile elements.

The diverse regions of these 20 plasmids were annotated as variable regions 1–3 (VR1–3) and resistance regions 1 and 2 (RR1 and RR2). A BLASTn search of VR1 showed 100% query coverage to eight chromosomes and two plasmids isolated from *P. aeruginosa* and the annotation of this unique region suggested that it did not contribute to resistance. VR2 of three pWTJH megaplasmids was almost absent from any of the other plasmids and the homology searches in GenBank showed that no highly related nucleotide sequences were found as coverage was <80%. Conversely, the VR3 flanked by IS*Ppu29* elements existed in most of the megaplasmids but pWTJH6. The resistance regions RR1 and RR2 were rich in transposases and integrases in proximity to various antimicrobial resistance (AMR) genes. Genes for mercury resistance operons(*merRTPCA*) were identified in pWTJH megaplasmids in RR2, which are commonly present on plasmids as part of transposons and exhibit a variety of arrangements (27). The β-lactamase genes in these plasmids were harbored in RR1. The *bla*_AFM_s were associated with the IS*CR29* elements which were responsible for dissemination. In addition, the core *bla*_KPC-2_ genetic platform IS*Kpn27-bla*_KPC-2_-IS*Kpn6* was associated with the mobile genetic elements Tn*1403*-like–ΔTn*6296*, while *bla*_IMP-45_ and the novel variant of *bla*_VIM_ in our study were cassette-borne in class 1 integron (Fig. S2; Fig. 2).

**Fig 3:**
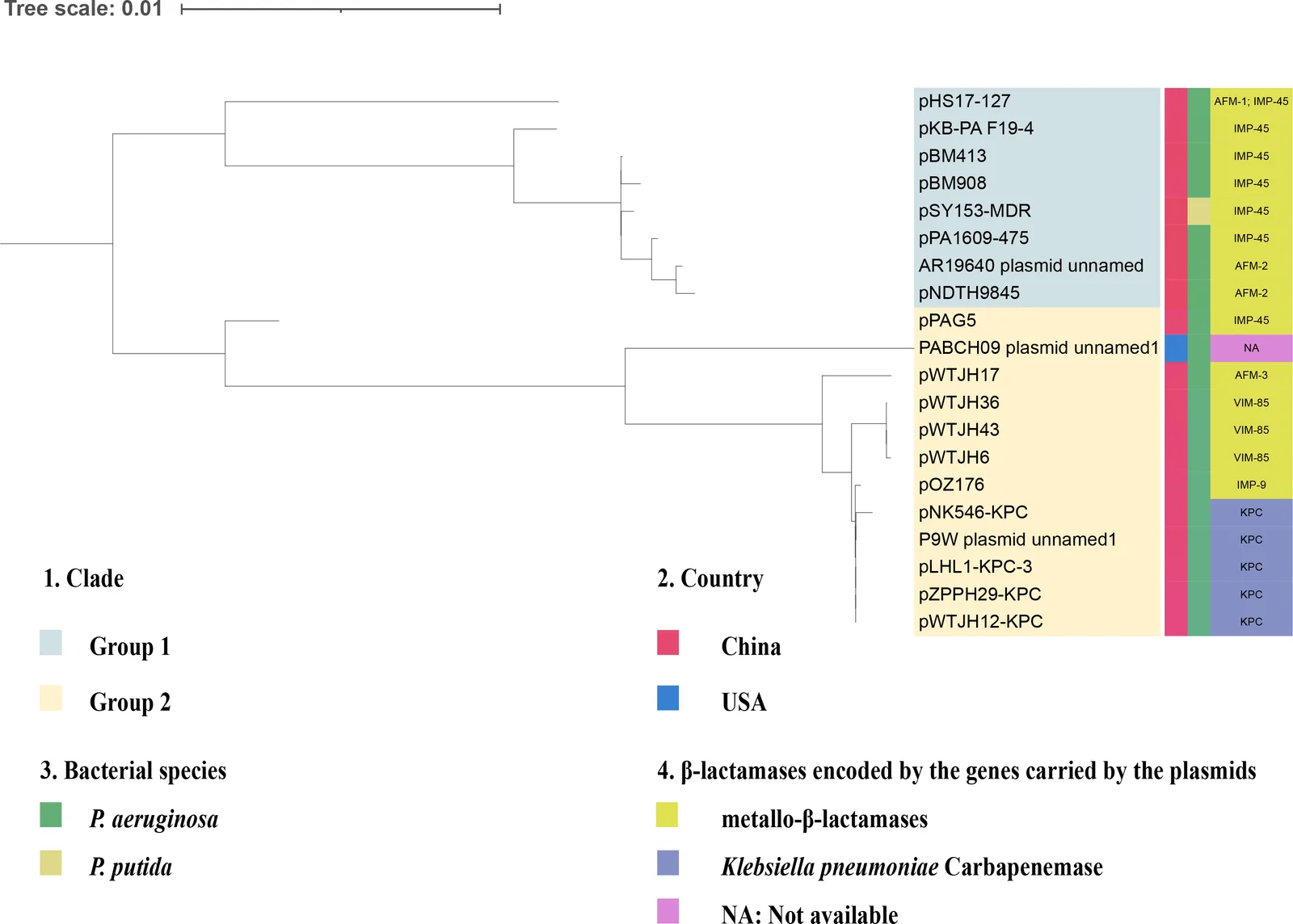
The phylogenetic tree based on the concatenated set of core genes (*n* = 370) displays the relationships of the three pWTJH megaplasmids and their 17 best hits in GenBank. The primary features of their hosts are indicated in various colors and more detailed information of the plasmids identiﬁed in GenBank is in Table S2.

To further understand the relationships between the 20 megaplasmids, we constructed a phylogenetic tree based on the nucleotide composition of core genes (*n* = 370) (Fig. 3). The 17 plasmids from Genbank were all carried by strains isolated from China except the plasmid unnamed one from PABCH09 (Genbank accession no. CP056096) which was obtained from the USA, suggesting that the members of IncP-2 megaplasmids were distributed geographically. The megaplasmids could be found in strains from two major groups. Plasmids pWTJH17 and pWTJH12-KPC, which were also isolated from WTJH, clustered close to the pWTJH megaplasmids in our study in the same clade. These observations suggested that nosocomial transmission of IncP-2 megaplasmids might have occurred in WTJH. The plasmid pOZ176 was closely related to the pWTJH megaplasmids. pOZ176 was also harbored by an MDR strain from China, sharing over 70% nucleotide coverages and over 99.8% identities to the pWTJH megaplasmids. The plasmid pOZ176 carried two integrons harboring *bla*_IMP-9_ and *bla*_OXA-10_, respectively, acting as vehicles for the spread of AMR genes regionally as the pWTJH megaplasmids did. Most of the plasmids were hosted by *P. aeruginosa* isolates while pSY153-MDR (Genbank accession no. KY883660) was obtained from a *P. putida* isolate, indicating transmission between species had occurred locally. Despite different species, pSY153-MDR carrying *bla*_IMP-45_ was closely related to the other plasmids associated with the *bla*_IMP-45_ genes. Similarly, a cluster of the megaplasmids harboring *bla*_KPC_s was observed, supporting the idea that the carrier megaplasmids acted as vehicles for the dissemination of AMR genes (Fig. 3).

### Conjugation, stability, and fitness cost of the pWTJH megaplasmids

To examine the transferability of pWTJH megaplasmids, all the *bla*_VIM85_-carrying pWTJH megaplasmids were performed conjugation with rifampin-resistant derivative of *P. aeruginosa* PAO1 as recipients. Transconjugants were obtained from WTJH36-PAO1 and WTJH43-PAO1 conjugation experiments with high efﬁciency (10^−2^ to 10^−3^ transconjugants per donor) but transfer of pWTJH6 from WTJH6 to PAO1 failed in more than three independent conjugation experiments. The stability of pWTJH36 and pWTJH43 in transconjugants was tested after continuous passage culture in non-selective media. We assessed the maintenance of resistance by calculating the ratio of the number of colonies on antibiotics-resistant plates to MH agar plates. No loss of resistance was detected, and plasmid maintenance was conﬁrmed using PCR by sequencing of *bla*_VIM-85_ and *repA*. To determine the fitness cost caused by the reception of megaplasmid, we analyzed the growth curves and found that both pWTJH36-bearing and pWTJH43-bearing PAO1 strains demonstrated impaired growth compared with plasmid-free PAO1, but the growth rate between megaplasmid-bearing PAO1 strains showed no difference (Fig. S3).

### Genetic characteristics and environment of the bla_VIM_ variants

We reconﬁrmed the sequences of *bla*_VIM-84_ and *bla*_VIM-85_ by PCR ampliﬁcation. Sequencing revealed that both *bla*_VIM-84_ and *bla*_VIM-85_ had a length of 801 bp, and they were highly similar to *bla*_VIM-24_ (GenBank accession no. HM855205) with a 99.88% and 99.75% nucleotide identity, respectively, according to BLASTn search. Both of them differed from *bla*_VIM-24_ by a nucleotide change (A179G), while *bla*_VIM-85_ obtained a further substitution at position 349 from G to A, resulting in one amino acid mutation (Gln60Arg) in VIM-84 and two amino acids replacements (Gln60Arg, Asp117Asn) in VIM-85 compared with VIM-24, respectively.

As expected, both *bla*_VIM-84_ and *bla*_VIM-85_ genes were embedded into class 1 integrons (Fig. 2). The *bla*_VIM-84_ gene was the second gene cassette of a class 1 integron with a *fosE* gene inserted upstream. The downstream cassettes were *aac (6′)-lb4*, *bla*_OXA-101_, and *ant1* genes successively. This integron was derived from In*2021* carrying *bla*_VIM-24_ but the *qacEΔ1* and *sul1* genes in the 3′ conserved segment (3′-CS) were deleted for the insertion of an IS*26* element. The genetic environment surrounding *bla*_VIM-85_ was *intI-bla*_VIM-85_-*aac (6′)-lb4-bla*_OXA-101_-*ant1-qacEΔ1-sul1* and the 3′-CS was inserted by a gene encoding GNAT family N-acetyltransferase which was clipped by the insertion of the transposon Tn*5393*. The integron carrying *bla*_VIM-85_ was embedded in the transposon Tn*1403* (GenBank accession no. AF313472.2) which was made up of the backbone containing genes *tnpA*, *tnpR*, and *orfABCD*, a class 1 integron, and Tn*5393*, the *tnpA* gene of which was disrupted by the insertion of the IS*66* family (28).

### Antimicrobial susceptibility of bacteria expressing *bla*_VIM-84_ *, bla*_VIM-85_ *,* and *bla*_VIM-24_

We first tested the antimicrobial susceptibility of the *E. coli* strains that we used for cloning. Even when either *bla*_VIM-84_, *bla*_VIM-85_, or *bla*_VIM-24_ were expressed in *E. coli*, the strain DH5α remained susceptible to aztreonam (Table 1). However, the MIC values of piperacillin, ceftazidime, imipenem, and meropenem in the presence of VIM-85 were four- to eightfold higher than VIM-24, while the MICs of cefepime were twofold lower. MICs of VIM-84 and VIM-85 expressions showed that VIM-85 conferred resistance equally well or rendered the strains more resistant to all antibiotics with the exception of aztreonam and ampicillin.

**TABLE 1:** MICs of β-lactam antibiotics for the *bla*_VIM-24_, *bla*_VIM-84_, and *bla*_VIM-85_ transformants

Antibiotic	MIC (mg/L)							
	*E. coli* (DH5α)				*P. aeruginosa* PAO1			
	pGK1900	pGK1900_VIM-24	pGK1900_VIM-84	pGK1900_VIM-85	pGK1900	pGK1900_VIM-24	pGK1900_VIM-84	pGK1900_VIM-85
Ampicillin	2	1,024	1,024	1,024	/	/	/	/
Piperacillin	<0.15	16	32	64	64	128	256	512
Cefepime	0.008	16	4	8	8	1,024	>1,024	>1,024
Ceftazidime	0.12	64	64	256	8	256	>1,024	>1,024
Piperacillin/TAZ	<0.5/4	64/4	64/4	128/4	16/4	128/4	512/4	512/4
Ceftazidime/AVI	0.0/4	128/4	128/4	256/4	2/4	1,024/4	>1,024/4	>1,024/4
Imipenem	0.12	4	4	16	8	64	256	256
Meropenem	0.016	2	4	16	2	128	256	256
Aztreonam	<0.03	0.06	<0.03	<0.03	8	4	4	4

^*a*^ TAZ, tazobactam (fixed concentration of 4 mg/L); AVI, avibactam (fixed concentration of 4 mg/L).

Next, we investigated the effects of the expression of *bla*_VIM_ in the original host *P. aeruginosa*, by introducing the plasmids into PAO1. We found that the expression of the *bla*_VIM_ proteins in PAO1 resulted in signiﬁcantly higher resistance levels to almost all antibiotics with the exception of aztreonam. The MICs of β-lactams, especially ceftazidime, piperacillin-tazobactam, and imipenem, were fourfold higher for VIM-84 and VIM-85 compared to VIM-24 when expressed in PAO1. However, the MICs of β-lactams, except piperacillin, were not higher for VIM-85 compared to VIM-84.

### Enzymatic activities

The expression of the VIM proteins in the bacteria allowed their survival due to the enzymatic inactivation of the compounds. To demonstrate the activity of the enzymes *in vitro*, we compared the hydrolytic activity of these two new VIM variants to other variants. Besides VIM-24, two additional VIM variants, VIM-2 and VIM-36, were included, since both VIM-84 and VIM-85 in our report belong to the VIM-2-like subgroup (Fig. S4), and VIM-36 differs from VIM-84 and VIM-85 by only one amino acid change (Leu205Arg) and two amino acids mutations (Leu205Arg and Asn117Asp). All of them were capable of hydrolyzing carbapenem antibiotics, with the highest activity toward imipenem, as demonstrated by the highest *k*_cat_/*K*_*m*_ values, compared to the other compounds (Table 2). This is consistent with the previous finding that imipenem is a suitable substrate for VIM-type enzymes (5). For both imipenem and meropenem, the enzymatic activities of VIM-84 and VIM-85 were slightly higher compared to VIM-24, VIM-2, and VIM-36. *K_m_* and *k*_cat_ values for VIM-2 and VIM-36 with piperacillin and cefepime could not be measured because the calculated *K_m_* values were much higher than the measured substrate concentrations. However, VIM-84 and VIM-85 hydrolyzed piperacillin at slightly higher levels than VIM-24, while hydrolytic efﬁciencies against cefepime were comparable among all three enzymes. A comparison of the kinetic parameters of VIM-85 with those of VIM-84 demonstrated that the former enzyme seemed not to be a more efﬁcient β-lactamase for all substrates tested.

**TABLE 2:** Kinetic parameters of VIM-84, VIM-85, VIM-24, VIM-2, and VIM-36

Substrate and parameter	Value of the parameter for:				
	VIM-84	VIM-85	VIM-24	VIM-2	VIM-36
**Piperacillin**					
*K*_*m*_ (μM)	101.74	59.59	68.86	NM^*a*^	NM^*a*^
*k*_cat_ (s^−1^)	202.99	131.87	78.66	NM^*a*^	NM^*a*^
*k*_cat_/*K*_*m*_ (μM^−1^ s^−1^)	2.92	2.33	1.18	NM^*a*^	NM^*a*^
**Cefepime**					
*K*_*m*_ (μM)	25.29	52.48	38.38	NM^*a*^	NM^*a*^
*k*_cat_ (s^−1^)	7.13	16.05	14.38	NM^*a*^	NM^*a*^
*k*_cat_/*K*_*m*_ (μM^−1^ s^−1^)	0.29	0.31	0.37	NM^*a*^	NM^*a*^
**Imipenem**					
*K*_*m*_ (μM)	6.16	9.30	9.15	10.31	15.73
*k*_cat_ (s^−1^)	21.75	26.99	11.35	11.12	12.48
*k*_cat_/*K*_*m*_ (μM^−1^ s^−1^)	3.60	2.90	1.26	1.10	0.83
**Meropenem**					
*K*_*m*_ (μM)	9.26	8.94	8.70	5.37	2.69
*k*_cat_ (s^−1^)	16.27	13.21	9.39	2.10	1.22
*k*_cat_/*K*_*m*_ (μM^−1^ s^−1^)	1.76	1.51	1.08	0.40	0.45

^*a*^ Not measured.

### Structural modeling of VIM-24, VIM-84, and VIM-85

**Fig 4:**
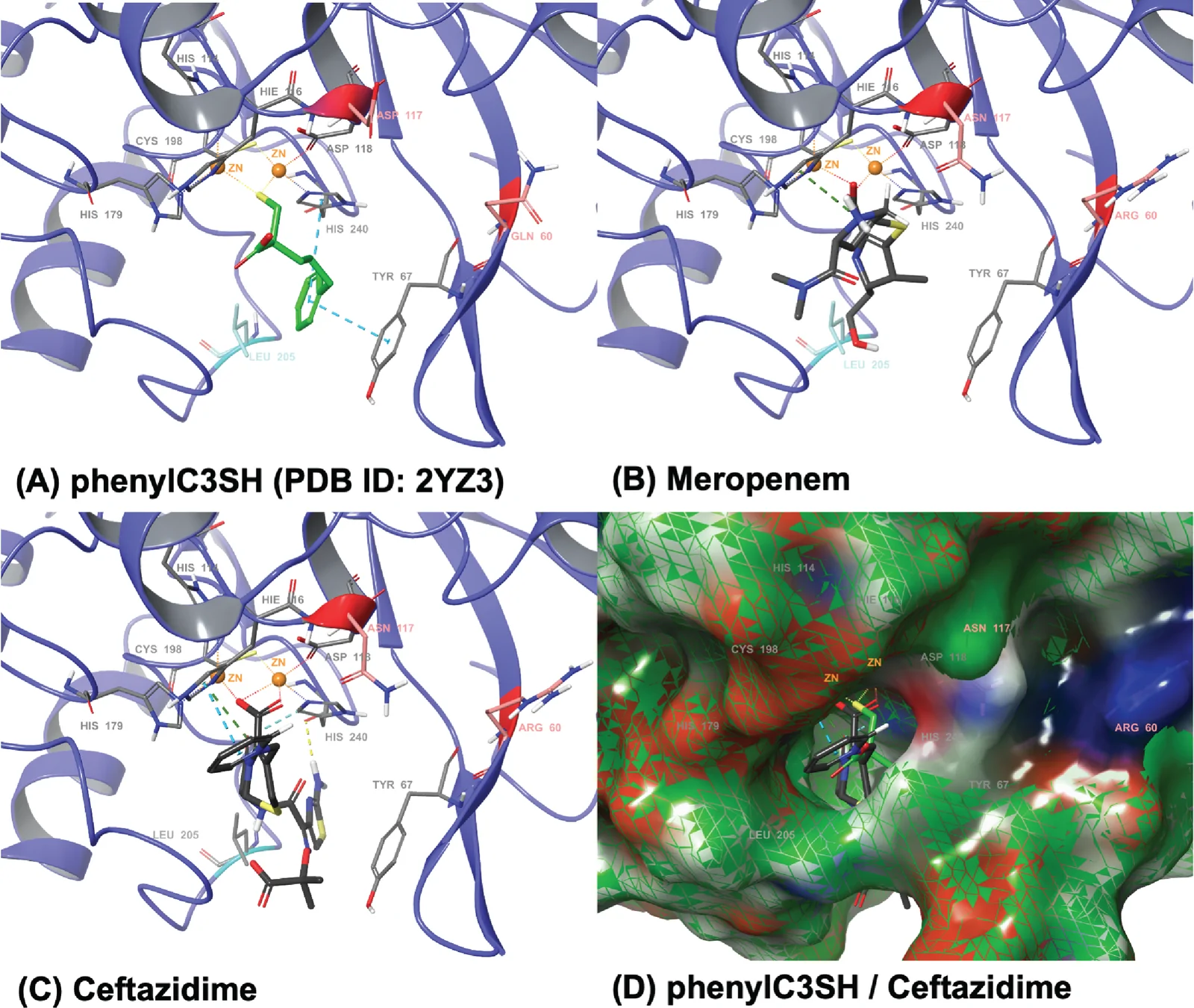
Binding mode analysis of phenylC3SH, meropenem, and ceftazidime bound to VIM. Panels (**A–C**) show the ribbon representation of ligand bound VIM-85 with nearby interaction residues, the carbon of the inhibitor is green while others are gray. The residues mutated from VIM24 and their corresponding position in the ribbon are colored red (VIM-85) and cyan (VIM-2). Zinc ions are colored gold. Panel (**D**) shows the superposed binding surface of VIM-85 (colored in residue charges, red for negative, and blue for positive) and VIM-24 (colored in green).

Having investigated the impact of the enzymes on the cells to inactivate the antibiotics, and after measuring the enzymatic activity of the purified proteins *in vitro*, we set out to understand the differences between the proteins on the structural level. We therefore obtained homology models of VIM-24, VIM-84, and VIM-85 (Fig. 4). Compared to VIM-24, VIM-84 contained a mutation in position 60 from Gln to Arg. One further amino acid replacement (Asp117Asn) was found in VIM-85 compared with VIM-84. Through *in silico* simulation, we found that the Q60R mutation was positioned in a hairpin loop linking β2 and β3 sheets. The D117N was located at the *R2* loop, while the R205L mutation compared to VIM-2 was located at the Ω loop. According to the modeling result, H116, H240, and Y67 were the key residues that interacted with lactams or inhibitors. The Arg in the Q60R mutation might provide a strong positive charged environment, facilitating the acylation of the cephalosporins by providing a proton thus increasing the hydrolysis capability of the enzyme toward the lactam. The mutations D117N and R205L were positioned outside the binding site of the pocket of the protein. An asparagine in position 117 would provide an additional amide group that resulted in additional hydrogen bonding possibilities. The formation of such bonds was observed in the docking complexes of the protein with meropenem and ceftazidime. As shown in Fig. 4B and C, the additional hydrogen bond between the carbonyl group of D117 and meropenem (2.21 Å) was longer than that of D117 and ceftazidime (2.86 Å). When R205L took place, the residue became smaller and electrically neutral, which reduced the distance but also charged restraints of the ligands to accommodate entry and binding to the substrate pocket of the enzyme. Similar observations have been reported before (29).

## DISCUSSION

Several clinical *P. aeruginosa* isolates in our study exhibited an extensive drug-resistant phenotype (30). While they remained susceptible to amikacin, resistance to gentamicin was observed, reﬂecting the existence of resistance mechanisms capable of counteracting the antibacterial activity of gentamicin without affecting amikacin effectiveness. Plasmid-encoded efﬂux pumps (MexCD-OprJ) are chromosomal resistance-nodulation-division (RND) family pumps mediating multidrug resistance in *Pseudomonas* spp. However, the gene clusters encoding MexCD-OprJ were observed to be encoded on plasmids in our study in pWTJH2 and pWTJH32, while two other groups reported a similar observation on unrelated plasmids in *P. putida* and *P. aeruginosa* (31, 32). These and our studies show that the plasmid-mediated transmission of these chromosomally located genes encoding RND-type efﬂux pumps has occurred and these resistance regions can be assembled dynamically in various plasmid backgrounds (7).

With the availability of long-read sequencing, an increasing number of related IncP-2-type megaplasmids carrying multiple cassette-borne carbapenemase genes have been investigated (7). The IncP-2 megaplasmids share a common core genetic backbone but are diverse with regard to their AMR gene proﬁles. Expanding gene content in conserved regions, such as genes involved in replication, segregation, and conjugation, improves the efficiency of vertical and horizontal transmission of megaplasmids (33). The beneficial traits in resistance regions provide adaptive advantages for their host and the associated mobile genetic elements play a role in the acquisition and dissemination of resistance genes. In general, the plasmids incorporate resistance genes via mobile elements and then disseminate within and beyond bacterial species (33). Our findings show that the characteristics of mobile elements highly correspond with the β-lactamase genes they transfer and the megaplasmids harboring the same resistance genes cluster together despite being found in different species. We found that the closely related megaplasmids of pWTJH43 occur mostly in clinical strains in China, and the plasmids from the same clinical setting cluster closely in the same group. Consistent with the phylogenetic analysis, stability and conjugation demonstrated high stability and high-efficiency transfer frequency of pWTJH36 and pWTJH43 megaplasmids, indicating that these megaplasmids could be locally distributed and responsible for the nosocomial transmission of AMR genes. However, the transfer of pWTJH6 failed since plasmid properties may vary across host genetic backgrounds (34). Although noncompetitive growth experiments showed the fitness cost of megaplasmid-bearing strains, plasmids could potentially survive at the population level by horizontal transmission if rates of conjugation were sufﬁciently high to partially offset the loss brought by the fitness costs (34).

A comparison of the MICs of PAO1 expressing *bla*_VIM-24_ and *bla*_VIM-84_ shows that *bla*_VIM-84_ confers similar or even higher resistance to all antibiotics except aztreonam, indicating VIM-84 is stronger or equally efficient in mediating resistance compared to VIM-24. In contrast, the MICs to most β-lactams, except piperacillin, are not higher for VIM-85 than those for VIM-84 expressed in PAO1, despite one more amino acid replacement (Asp117Asn) in VIM-85. All five recombinant VIM-type enzymes hydrolyzed all carbapenem antibiotics tested, but VIM-84 and VIM-85 show slightly greater enzymatic activities. D117N leads to additional hydrogen bonds but creates a weaker network than that of the R205L mutation due to the distance from the binding site within the pocket.

In conclusion, we detected two novel VIM-type MBLs, VIM-84 and VIM-85 which differed from VIM-24 by one amino acid mutation (Gln60Arg) and two amino acids replacements (Gln60Arg and Asp117Asn), respectively. Compared with VIM-24 expression, the MICs of *bla*_VIM-84_ and *bla*_VIM-85_ cloning strains show higher levels of resistance to β-lactams and the enzymatic activities of VIM-84 and VIM-85 demonstrate greater hydrolytic activities for most β-lactams. The IncP-2-type megaplasmids are distributed locally and appear to play an important role in the spread of AMR genes in the hospital setting.

## Data Availability

The nucleotide sequences reported in this study have been submitted to the GenBank databases under accession numbers CP104584–CP104585 (WTJH2 chromosome and pWTJH2), CP104586–CP104587 (WTJH6 chromosome and pWTJH6), CP104588– CP104589 (WTJH32 chromosome and pWTJH32), and CP104590–CP104591 (WTJH36 chromosome and pWTJH36). The Whole Genome Shotgun project of WTJH43 has been deposited at DDBJ/ENA/GenBank under the accession number JAODBV000000000. The accession numbers of blaVIM-84 and blaVIM-85 are ON688661 and ON688662, respectively.

## SUPPLEMENTAL MATERIAL

Supplemental figures and tables spectrum.01544-23-s0001.docx Tables S1 to S3 and Fig. S1 to S4.

ASM does not own the copyrights to Supplemental Material that may be linked to, or accessed through, an article. The authors have granted ASM a non-exclusive, world-wide license to publish the Supplemental Material files. Please contact the corresponding author directly for reuse.

## References

[1] Sleiman A , Fayad AGA , Banna H , Matar GM . 2021. Prevalence and molecular epidemiology of carbapenem-resistant gram-negative bacilli and their resistance determinants in the eastern mediterranean region over the last decade. J Glob Antimicrob Resist 25:209–221. doi:10.1016/j.jgar.2021.02.033 33812049

[2] Zhao W-H , Hu Z-Q . 2011. Epidemiology and genetics of VIM-type metallo-Β-lactamases in gram-negative bacilli. Future Microbiol 6:317–333. doi:10.2217/fmb.11.13 21449842

[3] Mauri C , Maraolo AE , Di Bella S , Luzzaro F , Principe L . 2021. The revival of aztreonam in combination with avibactam against metallo-Β-lactamase-producing gram-negatives: A systematic review of in vitro studies and clinical cases. Antibiotics (Basel) 10:1012. doi:10.3390/antibiotics10081012 34439062 PMC8388901

[4] Botelho J , Grosso F , Peixe L . 2019. Antibiotic resistance in Pseudomonas aeruginosa – mechanisms, epidemiology and evolution. Drug Resist Updat 44:100640. doi:10.1016/j.drup.2019.07.002 31492517

[5] Liu Z , Zhang R , Li W , Yang L , Liu D , Wang S , Shen J , Wang Y . 2019. Amino acid changes at the VIM-48 C-terminus result in increased carbapenem resistance, enzyme activity and protein stability. J Antimicrob Chemother 74:885–893. doi:10.1093/jac/dky536 30590504

[6] Yoon E-J , Jeong SH . 2021. Mobile carbapenemase genes in Pseudomonas aeruginosa. Front Microbiol 12:614058. doi:10.3389/fmicb.2021.614058 33679638 PMC7930500

[7] Cazares A , Moore MP , Hall JPJ , Wright LL , Grimes M , Emond-Rhéault J-G , Pongchaikul P , Santanirand P , Levesque RC , Fothergill JL , Winstanley C . 2020. A megaplasmid family driving dissemination of multidrug resistance in Pseudomonas. Nat Commun 11:1370. doi:10.1038/s41467-020-15081-7 32170080 PMC7070040

[8] Walker BJ , Abeel T , Shea T , Priest M , Abouelliel A , Sakthikumar S , Cuomo CA , Zeng Q , Wortman J , Young SK , Earl AM . 2014. Pilon: an integrated tool for comprehensive microbial variant detection and genome assembly improvement. PLoS One 9:e112963. doi:10.1371/journal.pone.0112963 25409509 PMC4237348

[9] Boratyn GM , Camacho C , Cooper PS , Coulouris G , Fong A , Ma N , Madden TL , Matten WT , McGinnis SD , Merezhuk Y , Raytselis Y , Sayers EW , Tao T , Ye J , Zaretskaya I . 2013. BLAST: a more efficient report with usability improvements. Nucleic Acids Res 41:W29–33. doi:10.1093/nar/gkt282 23609542 PMC3692093

[10] Zankari E , Hasman H , Cosentino S , Vestergaard M , Rasmussen S , Lund O , Aarestrup FM , Larsen MV . 2012. Identification of acquired antimicrobial resistance genes. J Antimicrob Chemother 67:2640–2644. doi:10.1093/jac/dks261 22782487 PMC3468078

[11] Tansirichaiya S , Rahman MA , Roberts AP . 2019. The transposon registry. Mob DNA 10:40. doi:10.1186/s13100-019-0182-3 31624505 PMC6785933

[12] Hua X , Liang Q , Deng M , He J , Wang M , Hong W , Wu J , Lu B , Leptihn S , Yu Y , Chen H . 2021. Bacant: a combination annotation server for bacterial DNA sequences to identify antibiotic resistance genes, integrons, and transposable elements. Front Microbiol 12:649969. doi:10.3389/fmicb.2021.649969 34367079 PMC8343408

[13] Alikhan N-F , Petty NK , Ben Zakour NL , Beatson SA . 2011. BLAST Ring Image Generator (BRIG): simple prokaryote genome comparisons. BMC Genomics 12:402. doi:10.1186/1471-2164-12-402 21824423 PMC3163573

[14] Tonkin-Hill G , MacAlasdair N , Ruis C , Weimann A , Horesh G , Lees JA , Gladstone RA , Lo S , Beaudoin C , Floto RA , Frost SDW , Corander J , Bentley SD , Parkhill J . 2020. Producing polished prokaryotic pangenomes with the panaroo pipeline. Genome Biol 21:180. doi:10.1186/s13059-020-02090-4 32698896 PMC7376924

[15] Nguyen L-T , Schmidt HA , von Haeseler A , Minh BQ . 2015. IQ-TREE: a fast and effective stochastic algorithm for estimating maximum-likelihood phylogenies. Mol Biol Evol 32:268–274. doi:10.1093/molbev/msu300 25371430 PMC4271533

[16] Letunic I , Bork P . 2019. Interactive Tree Of Life (iTOL) v4: recent updates and new developments. Nucleic Acids Res 47:W256–W259. doi:10.1093/nar/gkz239 30931475 PMC6602468

[17] Li Y , Zhu Y , Zhou W , Chen Z , Moran RA , Ke H , Feng Y , van Schaik W , Shen H , Ji J , Ruan Z , Hua X , Yu Y . 2022. Alcaligenes faecalis metallo-Β-lactamase in extensively drug-resistant Pseudomonas aeruginosa isolates. Clin Microbiol Infect 28:880. doi:10.1016/j.cmi.2021.11.012 34826621

[18] Ii JSL , Weinstein MP , Bobenchik AM , Campeau S , Cullen SK , Galas MF , Gold H , Humphries RM , Kirn TJ , Limbago B , Mathers AJ , Mazzulli T , Richter SS , Satlin M , Schuetz AN , Sharp S , Simner PJ. Performance standards for antimicrobial susceptibility testing 362.

[19] Laraki N , Franceschini N , Rossolini GM , Santucci P , Meunier C , de Pauw E , Amicosante G , Frère JM , Galleni M . 1999. Biochemical characterization of the Pseudomonas aeruginosa 101/1477 metallo-beta-lactamase IMP-1 produced by Escherichia coli . Antimicrob Agents Chemother 43:902–906. doi:10.1128/AAC.43.4.902 10103197 PMC89223

[20] Madhavi Sastry G , Adzhigirey M , Day T , Annabhimoju R , Sherman W . 2013. Protein and ligand preparation: parameters, protocols, and influence on virtual screening enrichments. J Comput Aided Mol Des 27:221–234. doi:10.1007/s10822-013-9644-8 23579614

[21] Harder E , Damm W , Maple J , Wu C , Reboul M , Xiang JY , Wang L , Lupyan D , Dahlgren MK , Knight JL , Kaus JW , Cerutti DS , Krilov G , Jorgensen WL , Abel R , Friesner RA . 2016. OPLS3: a force field providing broad coverage of drug-like small molecules and proteins. J Chem Theory Comput 12:281–296. doi:10.1021/acs.jctc.5b00864 26584231

[22] Yeo CC , Tham JM , Kwong SM , Yiin S , Poh CL . 1998. Tn5563, a transposon encoding putative mercuric ion transport proteins located on plasmid pRA2 of Pseudomonas alcaligenes *.* FEMS Microbiol Lett 165:253–260. doi:10.1111/j.1574-6968.1998.tb13154.x 9742696

[23] Xiong J , Alexander DC , Ma JH , Déraspe M , Low DE , Jamieson FB , Roy PH . 2013. Complete sequence of pOZ176, a 500-kilobase IncP-2 plasmid encoding IMP-9-mediated carbapenem resistance, from outbreak isolate Pseudomonas aeruginosa 96. Antimicrob Agents Chemother 57:3775–3782. doi:10.1128/AAC.00423-13 23716048 PMC3719692

[24] Zhang X , Wang L , Li D , Li P , Yuan L , Yang F , Guo Q , Wang M . 2021. An IncP-2 plasmid sublineage associated with dissemination of bla_IMP-45_ among carbapenem-resistant Pseudomonas aeruginosa. Emerg Microbes Infect 10:442–449. doi:10.1080/22221751.2021.1894903 33620296 PMC7971254

[25] Burrows LL . 2012. Pseudomonas aeruginosa twitching motility: type IV pili in action. Annu Rev Microbiol 66:493–520. doi:10.1146/annurev-micro-092611-150055 22746331

[26] Smillie C , Garcillán-Barcia MP , Francia MV , Rocha EPC , de la Cruz F . 2010. Mobility of plasmids. Microbiol Mol Biol Rev 74:434–452. doi:10.1128/MMBR.00020-10 20805406 PMC2937521

[27] Nascimento AMA , Chartone-Souza E . 2003. Operon mer: bacterial resistance to mercury and potential for bioremediation of contaminated environments. Genet Mol Res 2:92–101. 12917805

[28] Stokes HW , Elbourne LDH , Hall RM . 2007. Tn1403, a multiple-antibiotic resistance transposon made up of three distinct transposons. Antimicrob Agents Chemother 51:1827–1829. doi:10.1128/AAC.01279-06 17261631 PMC1855573

[29] Mojica MF , Mahler SG , Bethel CR , Taracila MA , Kosmopoulou M , Papp-Wallace KM , Llarrull LI , Wilson BM , Marshall SH , Wallace CJ , Villegas MV , Harris ME , Vila AJ , Spencer J , Bonomo RA . 2015. Exploring the role of residue 228 in substrate and inhibitor recognition by VIM metallo-Β-lactamases. Biochemistry 54:3183–3196. doi:10.1021/acs.biochem.5b00106 25915520 PMC4700511

[30] Magiorakos A-P , Srinivasan A , Carey RB , Carmeli Y , Falagas ME , Giske CG , Harbarth S , Hindler JF , Kahlmeter G , Olsson-Liljequist B , Paterson DL , Rice LB , Stelling J , Struelens MJ , Vatopoulos A , Weber JT , Monnet DL . 2012. Multidrug-resistant, extensively drug-resistant and pandrug-resistant bacteria: an international expert proposal for interim standard definitions for acquired resistance. Clin Microbiol Infect 18:268–281. doi:10.1111/j.1469-0691.2011.03570.x 21793988

[31] Yuan M , Chen H , Zhu X , Feng J , Zhan Z , Zhang D , Chen X , Zhao X , Lu J , Xu J , Zhou D , Li J . 2017. pSY153-MDR,A p12969-DIM-related MEGA plasmid carrying bla_IMP-45_ and armA, from clinical Pseudomonas Putida. Oncotarget 8. doi:10.18632/oncotarget.19496 PMC5620268 28978128

[32] Liu J , Yang L , Chen D , Peters BM , Li L , Li B , Xu Z , Shirtliff ME . 2018. Complete sequence of pBM413, a novel multidrug resistance Megaplasmid carrying qnrVC6 and bla_IMP-45_ from Pseudomonas aeruginosa. Int J Antimicrob Agents 51:145–150. doi:10.1016/j.ijantimicag.2017.09.008 28923459

[33] Hall JPJ , Botelho J , Cazares A , Baltrus DA . 2022. What makes a megaplasmid? Philos Trans R Soc Lond B Biol Sci 377:20200472. doi:10.1098/rstb.2020.0472 34839707 PMC8628078

[34] Brockhurst MA , Harrison E . 2022. Ecological and evolutionary solutions to the plasmid paradox. Trends Microbiol 30:534–543. doi:10.1016/j.tim.2021.11.001 34848115

