# Supplementary material for: Characterization of two novel VIM-type metallo-β-lactamases, VIM-84 and VIM-85, associated with the spread of IncP-2 megaplasmids in Pseudomonas aeruginosa
Source: Microbiol Spectr. 2023 Sep 14;11(5):e01544-23. doi: 10.1128/spectrum.01544-23 (PMC10580930; doi:10.1128/spectrum.01544-23)
Supplement: Supplemental figures and tables — Tables S1 to S3 and Fig. S1 to S4. [file spectrum.01544-23-s0001.docx]

| Table S1. Primers used in this study | |
| --- | --- |
| Genes | Primer Sequences (5’→3’) |
| Sequence of predicted promoter + *bla*_VIM-84_ | F: ACGACTCACTATAGGGCGAATGCCGTAGAAGAACAGCAAG |
|  | R: CGAGGGCAGAGCCATGAGAAGCAACTTCATGTTATGCCGC |
| Sequence of predicted promoter + *bla*_VIM-85_ | F1: ACGACTCACTATAGGGCGAATGCCGTAGAAGAACAGCAAG |
|  | R1: AAGTGCGTGGAGACTGCACGCGTTACAGGAAGTC |
|  | F2: TGCAGTCTCCACGCACTTTCATAACGACCGCGTCG |
|  | R2: ACGACTCACTATAGGGCGAATGCCGTAGAAGAACAGCAAG |
| Sequence of predicted promoter + *bla*_VIM-24_ | F1: ACGACTCACTATAGGGCGAATGCCGTAGAAGAACAGCAAG |
|  | R1: TGCGATATGCGACCAAACACCATCGGCAATCTGGTAAAG |
|  | F2: GTGTTTGGTCGCATATCGCAACGCAGTCGTTTGATGGCGC |
|  | R2: CGAGGGCAGAGCCATGAGAAGCAACTTCATGTTATGCCGC |
| pGK1900 | F: TTCTCATGGCTCTGCCCTC |
|  | R: TTCGCCCTATAGTGAGTCGT |
| ORFs coding for VIM-2, VIM-24, VIM-84, VIM-85 without signal peptide regions | F: GAAAACCTGTATTTTCAGGGCAGTCCGCTCGCTTTTTCCG |
|  | R: CGCGGCACCAGGCCGCTGCTCTACTCAACGACTGAGCGATTTGTG |
| ORFs coding for VIM-36 without signal peptide regions | F1: GAAAACCTGTATTTTCAGGGCAGTCCGCTCGCTTTTTCCG |
|  | R1: GACAACTCATAAATCGCACAACCACCATAGAGCACACTCG |
|  | F2: GTGCGATTTATGAGTTGTCACGCACGTCTGCGGGGAACG |
|  | R2: CGCGGCACCAGGCCGCTGCTCTACTCAACGACTGAGCGATTTGTG |
| pET28a | F: AGCAGCGGCCTGGTGCCGCGCGGCAGCCATATG |
|  | R: GCCCTGAAAATACAGGTTTTCGTGATGATGATGATGATGGCTGCTGCCCATGGT |
| *repA* | F: CAAGCCTTTTCCGTAGGAGC |
|  | R: TAAAGGTGCTAGTGCCCCAC |

| Table S2. Gene information and MICs of the clinical bacterial strains | | | | | | | | | | | | | | | | | |
| --- | --- | --- | --- | --- | --- | --- | --- | --- | --- | --- | --- | --- | --- | --- | --- | --- | --- |
| Isolate | **MLST** | **VIM-24 variants** | **Genome characteristics** | | | **MICs(mg/L)** | | | | | | | | | | | |
|  |  |  | Molecule | Size (bp) | Antibiotic resistance genes | PIP | FEP | CAZ | PTZ | CZA | IMP | MEM | AZT | AK | GM | LEV | COL |
| WTJH2 | ST179 | VIM-84  (Q60R) | chromosome | 6949333 | *bla*_OXA-396_*, crpP, catB7, aph(3')-IIb, bla*_PDC-374_*, fosA* | **512** | **1024** | **>1024** | **256/4** | **>1024/4** | **512** | **1024** | 16 | 16 | **512** | 1 | 0.5 |
|  |  |  | pWTJH2 | 103681 | *fosE, bla*_VIM-84_*, aac(6')-Ib4, bla*_OXA-101_*, aadA1* |  |  |  |  |  |  |  |  |  |  |  |  |
| WTJH32 | ST179 | VIM-84  (Q60R) | chromosome | 6942888 | *bla*_OXA-396_*, crpP, catB7, aph(3')-IIb, bla*_PDC-37_*_4_, fosA* | **1024** | **1024** | **1024** | **512/4** | **>1024/4** | **512** | **1024** | 16 | 16 | **512** | 1 | 0.5 |
|  |  |  | pWTJH32 | 109939 | *fosE, bla*_VIM-84_*, aac(6')-Ib4, bla*_OXA-101_*, aadA1* |  |  |  |  |  |  |  |  |  |  |  |  |
| WTJH6 | ST360 | VIM-85  (Q60R, D117N) | chromosome | 6757988 | *aph(3')-IIb, bla*_PDC-374_*, bla*_OXA-903_*, catB7, crpP, fosA* | **256** | **256** | **1024** | **128/4** | **>1024/4** | **256** | **64** | 4 | 4 | **32** | <0.5 | 0.5 |
|  |  |  | pWTJH6 | 426499 | *bla*_VIM-85_*, aac(6')-Ib4, bla*_OXA-101_*, aadA1, sul1, aph(3'')-Ib, aph(6)-Id* |  |  |  |  |  |  |  |  |  |  |  |  |
| WTJH36 | ST179 | VIM-85  (Q60R, D117N) | chromosome | 7094521 | *bla*_OXA-396_*, catB7, aph(3')-IIb, bla*_PDC-374_*, fosA, crpP* | **512** | **1024** | **>1024** | **512/4** | **>1024/4** | **64** | **16** | 8 | 4 | **32** | <0.5 | 0.5 |
|  |  |  | pWTJH36 | 462066 | *bla*_VIM-85_*, aac(6')-Ib4, bla*_OXA-101_*, aadA1, sul1, aph(3'')-Ib, aph(6)-Id* |  |  |  |  |  |  |  |  |  |  |  |  |
| WTJH43 | ST179 | VIM-85  (Q60R, D117N) | chromosome | 7102711 | *bla*_OXA-396_*, catB7, aph(3')-IIb, bla*_PDC-374_*, fosA, crpP* | **512** | **512** | **>1024** | **512/4** | **>1024/4** | **64** | **32** | 16 | 4 | **32** | <0.5 | 0.5 |
|  |  |  | pWTJH43 | 462097 | *bla*_VIM-85_*, aac(6')-Ib4, bla*_OXA-101_*, aadA1, sul1, aph(3'')-Ib, aph(6)-Id* |  |  |  |  |  |  |  |  |  |  |  |  |
| PIP, piperacillin; FEP, cefepime; CAZ, ceftazidime; PTZ, piperacillin-tazobactam; CZA, ceftazidime-avibactam; IMP, imipenem; MEM, meropenem; AZT, aztreonam; AK, amikacin; GM, gentamicin; LEV, levofloxacin; COL, colistin. | | | | | | | | | | | | | | | | | |

| Table S3. Characteristics of complete megaplasmids from Genbank | | | | | | | |  |  |
| --- | --- | --- | --- | --- | --- | --- | --- | --- | --- |
| Plasmid | Bacterial species | Strain | Size | Country | Source | β-lactamases | Accession number | Identity* | Reference |
| pHS17-127 | *P. aeruginosa* | HS17-127 | 486963 | China, Shanghai | respiratory tract | AFM-1; IMP-45 | CP061377 | 98.76% | (1) |
| pKB-PA_F19-4 | *P. aeruginosa* | KB-PA_F19 | 412187 | China, Kunming | skin secretions | IMP-45 | CP086014 | 98.75% | Not available |
| pBM413 | *P. aeruginosa* | PA121617 | 423,017 | China, Guangzhou | respiratory tract | IMP-45 | CP016215 | 98.76% | (2) |
| pBM908 | *P. aeruginosa* | PA298 | 395774 | China, Guangzhou | feces | IMP-45 | CP040126 | 99.02% | Not available |
| pSY153-MDR | *P. putida* | SY-153 | 468,170 | China, Beijing | urinary tract | IMP-45 | KY883660 | 97.77% | (3) |
| pPA1609-475 | *P. aeruginosa* | PA1609 | 475129 | China, Wenzhou | NA | IMP-45 | CP090650 | 98.72% | Not available |
| unnamed | *P. aeruginosa* | AR19640 | 495621 | China, Hangzhou | rectal swab | AFM-2 | CP095921 | 98.57% | Not available |
| pNDTH9845 | *P. aeruginosa* | NDTH9845 | 463517 | China, Nanjing | urinary tract | AFM-2 | CP073081 | 98.72% | (4) |
| pPAG5 | *P. aeruginosa* | PAG5 | 513322 | China, Shaanxi | urinary tract | IMP-45 | CP045003 | 99.10% | (5) |
| unnamed1 | *P. aeruginosa* | PABCH09 | 510959 | USA | respiratory tract | NA | CP056096 | 99.85% | Not available |
| pWTJH17 | *P. aeruginosa* | WTJH17 | 436486 | China, Wuhan | bloodstream | AFM-3 | CP073083 | 99.85% | (4) |
| pOZ176 | *P. aeruginosa* | PA96 | 500,839 | China, Guangzhou | respiratory tract | IMP-9 | KC543497 | 99.89% | (6) |
| pNK546-KPC | *P. aeruginosa* | PAB546 | 475027 | China, Tianjin | NA | KPC-2 | MN433457 | 99.91% | Not available |
| unnamed 1 | *P. aeruginosa* | P9W | 475028 | China, Tianjin | burn wound | KPC-2 | CP081203 | 99.91% | Not available |
| pLHL1-KPC-3 | *P. aeruginosa* | LHL | 394987 | China, Zhengzhou | respiratory tract | KPC-3 | CP099961 | 99.91% | (7) |
| pZPPH29-KPC | *P. aeruginosa* | ZPPH29 | 397554 | China, Hangzhou | burn wound | KPC-2 | CP077978 | 99.90% | (8) |
| pWTJH12-KPC | *P. aeruginosa* | WTJH12 | 396963 | China, Wuhan | NA | KPC-2 | CP064404 | 99.90% | (8) |
| * The identities of complete nucleotide sequences of megaplasmids from GenBank compared to pWTJH43. | | | | | | | | | |


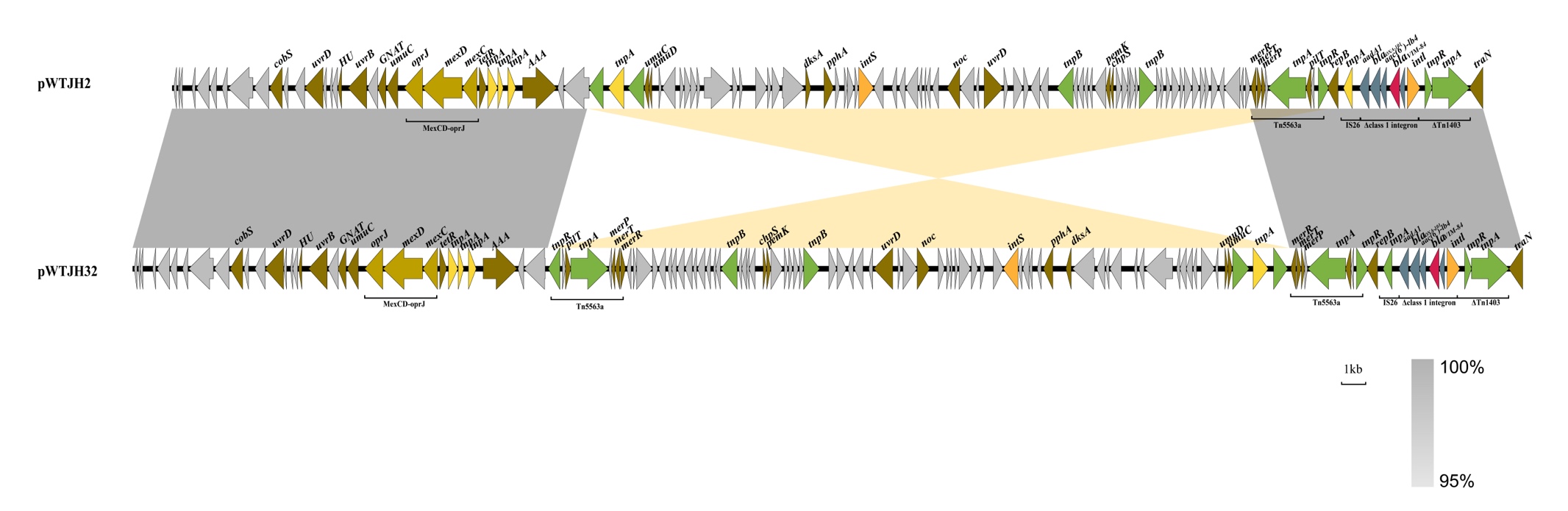


**Figure S1.** The pairwise comparison of pWTJH2 and pWTJH32. Shaded regions denote nucleotide identity (95%-100%). Red arrows denote the genes *bla*_VIM_ and the other antibiotic resistance genes are denoted by dark blue arrows. Yellow, green and light blue arrows denote structures of mobile elements.


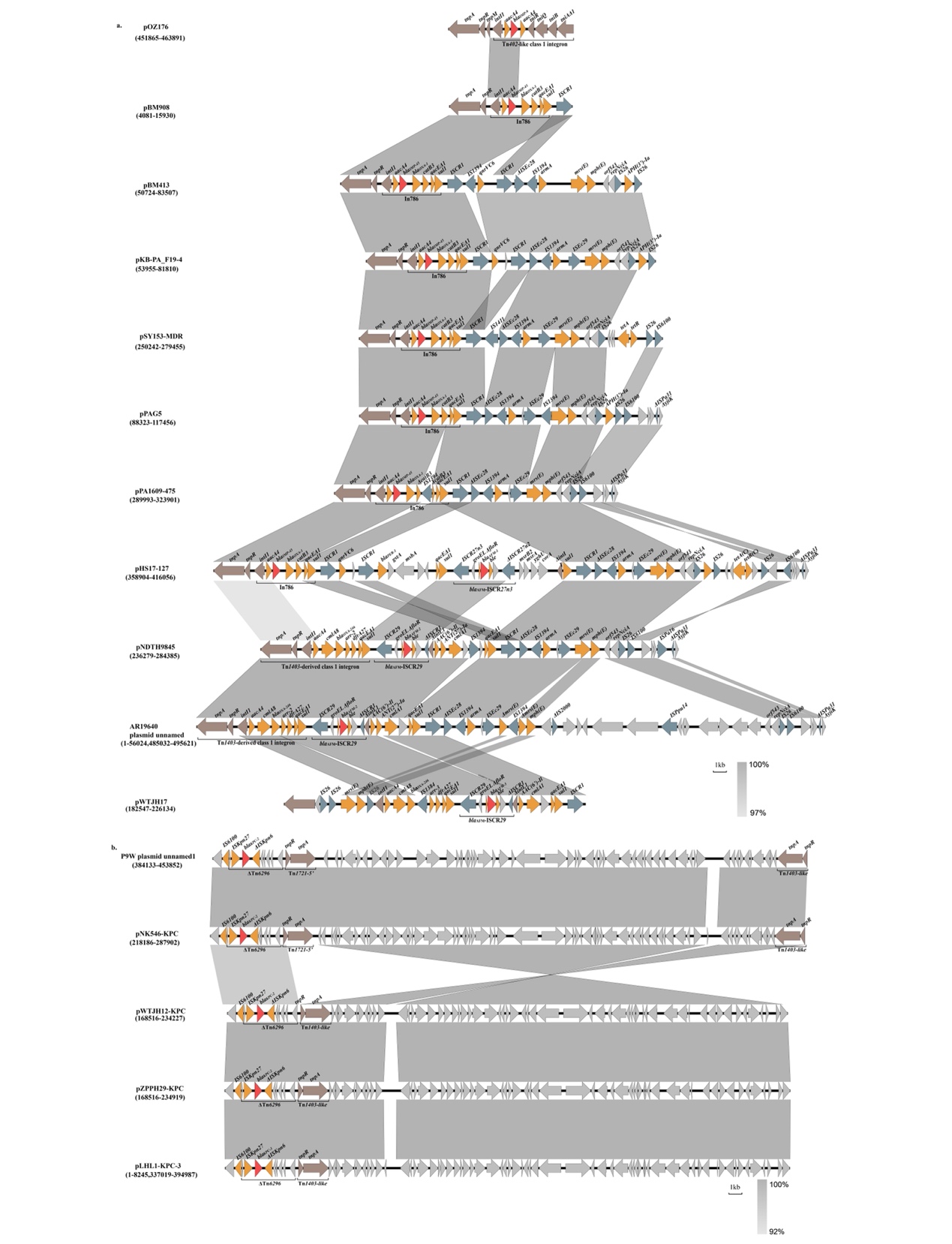


**Figure S2.** The pairwise comparison of the genetic environment of the β-lactamase genes in megaplasmids from Genbank. Shaded regions denote nucleotide identity (95%-100% in Figure S3a, 92%-100% in Figure S3 b). The accession numbers and references of these plasmids are listed in Table S3.


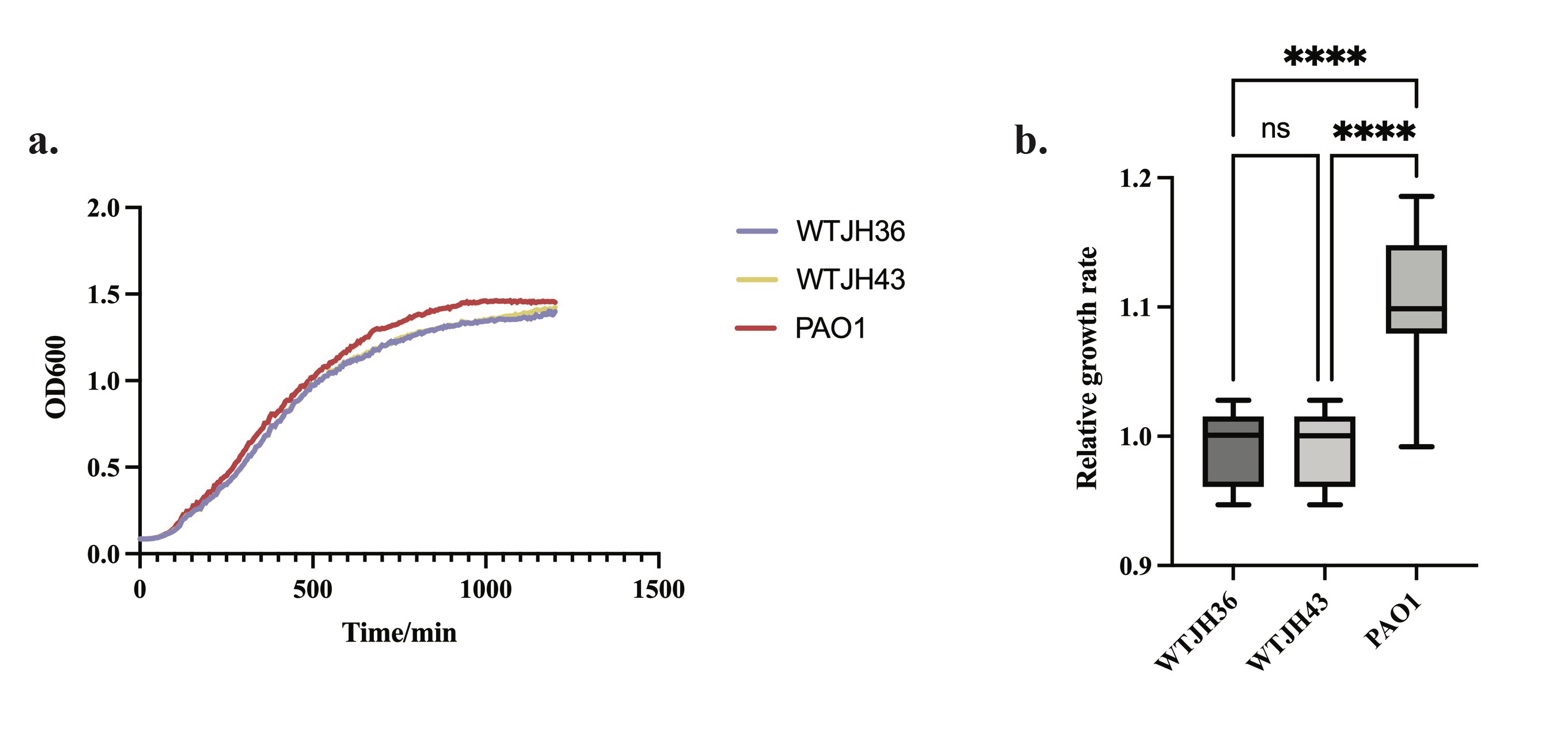


**Figure S3.** A growth kinetics of WTIH36, WTJH43 and PAO1. (a) Growth curves of WTIH36, WTJH43 and PAO1 in MH broth medium without antibiotics. (b) The relative growth rate of WTIH36, WTJH43 and PAO1 are expressed as means ± standard deviations.


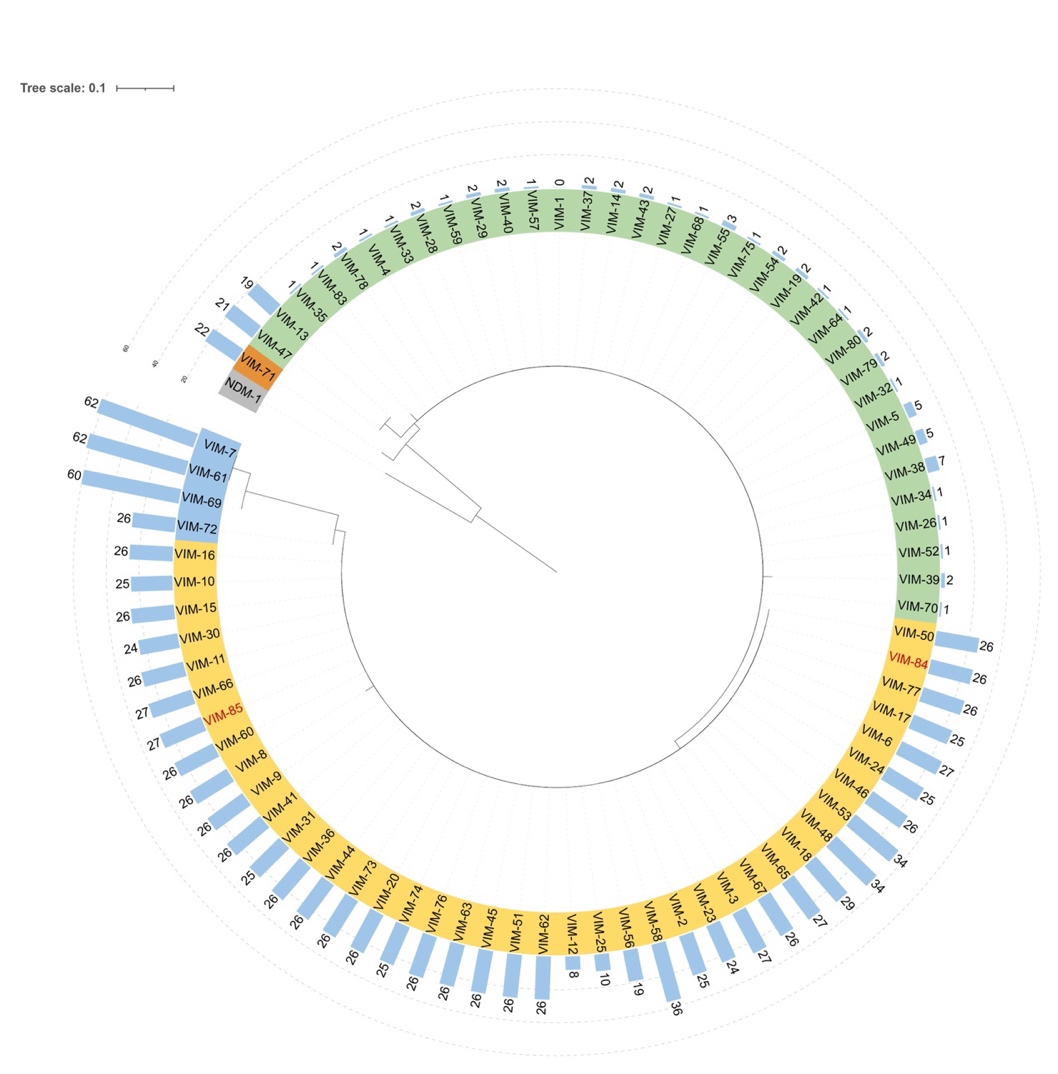


**Figure S4.** The phylogeny of the VIM family. Labels of the phylogenetic tree are highlighted in different colors representing different subgroups. Mutation numbers of amino acid sequences of VIM variants compared with VIM-1 are represented by the bar graph on the outmost circle. Amino acid sequences were obtained from the Genbank database.

**References**

1. Zhang X, Wang L, Li D, Wang C, Guo Q, Wang M. 2021. Characterization of the novel plasmid-encoded MBL gene *bla* AFM-1, integrated into a *bla* IMP-45-bearing transposon Tn *6485e* in a carbapenem-resistant *Pseudomonas aeruginosa* clinical isolate. Journal of Antimicrobial Chemotherapy 77:83–88.

2. Liu J, Yang L, Chen D, Peters BM, Li L, Li B, Xu Z, Shirtliff ME. 2018. Complete sequence of pBM413, a novel multidrug resistance megaplasmid carrying qnrVC6 and bla IMP-45 from pseudomonas aeruginosa. International Journal of Antimicrobial Agents 51:145–150.

3. Yuan M, Chen H, Zhu X, Feng J, Zhan Z, Zhang D, Chen X, Zhao X, Lu J, Xu J, Zhou D, Li J. 2017. pSY153-MDR, a p12969-DIM-related mega plasmid carrying *bla* IMP-45 and *armA* , from clinical *Pseudomonas putida*. Oncotarget 8:68439–68447.

4. Li Y, Zhu Y, Zhou W, Chen Z, Moran RA, Ke H, Feng Y, van Schaik W, Shen H, Ji J, Ruan Z, Hua X, Yu Y. 2022. Alcaligenes faecalis metallo-β-lactamase in extensively drug-resistant Pseudomonas aeruginosa isolates. Clin Microbiol Infect 28:880.e1-880.e8.

5. Li M, Guan C, Song G, Gao X, Yang W, Wang T, Zhang Y. 2022. Characterization of a Conjugative Multidrug Resistance IncP-2 Megaplasmid, pPAG5, from a Clinical Pseudomonas aeruginosa Isolate. Microbiol Spectr 10:e01992-21.

6. Xiong J, Alexander DC, Ma JH, Déraspe M, Low DE, Jamieson FB, Roy PH. 2013. Complete Sequence of pOZ176, a 500-Kilobase IncP-2 Plasmid Encoding IMP-9-Mediated Carbapenem Resistance, from Outbreak Isolate Pseudomonas aeruginosa 96. Antimicrob Agents Chemother 57:3775–3782.

7. Ge H, Qiao J, Zheng J, Xu H, Liu R, Zhao J, Chen R, Li C, Guo X, Zheng B. 2023. Emergence and clonal dissemination of KPC-3-producing Pseudomonas aeruginosa in China with an IncP-2 megaplasmid. Ann Clin Microbiol Antimicrob 22:31.

8. Zhu Y, Chen J, Shen H, Chen Z, Yang Q, Zhu J, Li X, Yang Q, Zhao F, Ji J, Cai H, Li Y, Zhang L, Leptihn S, Hua X, Yu Y. 2021. Emergence of Ceftazidime- and Avibactam-Resistant Klebsiella pneumoniae Carbapenemase-Producing Pseudomonas aeruginosa in China. mSystems 6:e00787-21.
